# The complete mitochondrial genome of a mangrove associated plant: *Sesuvium portulacastrum* and its phylogenetic implications

**DOI:** 10.1080/23802359.2019.1698982

**Published:** 2020-08-03

**Authors:** Renmao Li, Xianya Wei, Yun Wang, Ying Zhang

**Affiliations:** a Life Science and Technology School, Lingnan Normal University, Zhanjiang, China; b Chengdu Agricultural College, Chengdu, China

**Keywords:** *Sesuvium portulacastrum*, Aizoaceae, mitochondrial genome, Phylogenomic tree

## Abstract

The complete mitochondrial genome of mangrove associated plant: *Sesuvium portulacastrum* was analyzed in this paper, which is the first for the genus within the family Aizoaceae. The mitogenome sequence is 392,221 bp in length containing six ribosomal RNA genes, 27 transfer RNA genes, and 36 protein-coding genes. Gene *nad*1, *nad*2 and *nad*5 are the trans-splicing genes. One intron is found in gene *ccmFc*, two introns are found in genes *nad4* and *rps3*, and four introns are found in gene *nad7*. Phylogenetic analysis using the maximum likelihood method positioned *S. portulacastrum* within the monophyletic clades of the family Aizoaceae.

*Sesuvium portulacastrum* belonging to Aizoaceae species, is a perennial, dichotomous, dicotyledonous halophyte plant, and often grows in coastal and inland sandy soils around the world (Yi et al. 2014; Chang et al. 2016). The species has the ability to be propagated by salt-tolerant vegetative fragments as well as to its tolerance of salt spray, sand scouring and burial, high substrate temperatures (Rabhi et al. 2010). It was found to be efficient in bio reclamation of salt – affected soils as a result of its aptitude to produce high biomass and to accumulate enormous sodium quantities within its shoots (Munns 2002). Mitochondrial genome based phylogenetic analysis would improve our understanding of the evolutionary relationship of this plant under tidal habitat. In this study, we sequenced and analyzed the complete mitochondrial DNA sequence of *S. portulacastrum*. This is the first complete mitogenome within the family Aizoaceae.

Fresh leaves were collected from three individual of *S. portulacastrum* in XinYing Mangrove Natural Garden, Hainan Island (N19°30′, E109°30′), China. The total genomic DNA was extracted from ten mixed fresh leaves of *S. portulacastrum* by using the modified CTAB method (Doyle 1987) in the laboratory of Lingnan Normal University. Further, the specimen was also stored in the herbarium of Lingnan Normal University (No. Sp20190624-001). Genome sequencing was performed on an Illumina Hiseq X Ten platform with paired-end reads of 150 bp. In total, 47.8 Gb short sequence data with Q20 was 97.16% was obtained. The remaining high-quality reads were used to assemble the mitogenome using NOVOPlasty (Dierckxsens et al. 2017), where *Beta macrocarpa* (GenBank accession FQ378026.1) used as the seed sequence. The genes annotation in the mitogenome was doing with SPAdes v.3.9.0 (Bankevich et al. 2012) and some genes were annotated manually. The accession number in Genbank is MN683736. Eight mitogenome sequences in Caryophyllales were aligned including *S. portulacastrum*, and *Arabidopsis thaliana* was used as the out-group species. Phylogenetic analysis using the maximum likelihood algorithm was conducted with Mega X (Kumar et al. 2018).

The mitogenome of *S. portulacastrum* is 392,221bp in length with GC content of 42.17%, which contains six ribosomal RNA genes (2 *rrn16*, 2 *rrn12* and 2 *rrn5*), 27 transfer RNA genes, and 36 protein-coding genes. Among those genes, *ccmFc* contains one intron, *nad4* and *rps3* contain two introns, and *nad7* contain four introns. Genes *nad1*, *nad2*, and *nad5* are trans-splicing genes. Phylogenetic analysis with other seven plant mitogenomes showed that *S. portulacastrum* is sister to a clade containing four species in Caryophyllales (Figure 1). The useful genomic resources for characterization of genetic diversity of *S. portulacastrum* by the mitogenome will help for the study of evolution mechanism.

**Figure 1.**
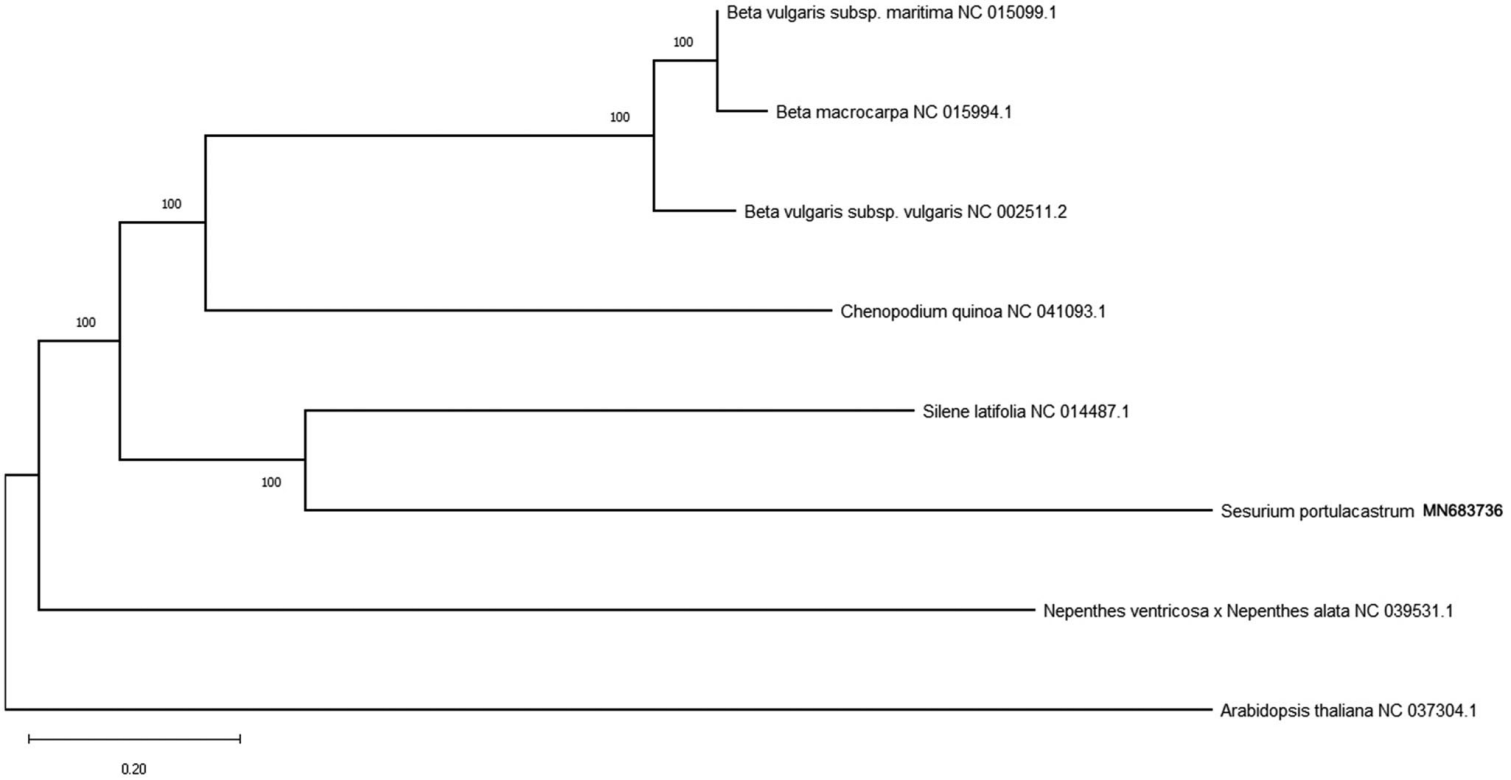
Maximum likelihood tree based on the sequences of eight complete mitogenomes. Numbers in the nodes were bootstrap values from 1000 replicates. Scale in substitutions per site.
